# Early versus late parenteral nutrition in ICU patients: cost analysis of the EPaNIC trial

**DOI:** 10.1186/cc11361

**Published:** 2012-05-25

**Authors:** Simon Vanderheyden, Michael P Casaer, Katrien Kesteloot, Steven Simoens, Thomas De Rijdt, Guido Peers, Pieter J Wouters, Jocelijn Coenegrachts, Tine Grieten, Katleen Polders, Ann Maes, Alexander Wilmer, Jasperina Dubois, Greet Van den Berghe, Dieter Mesotten

**Affiliations:** 1 Laboratory & Department of Intensive Care Medicine, University Hospitals Leuven, Herestraat 49, Leuven, B-3000, Belgium; 2 Finance & Accounting Department of the University Hospitals Leuven, Herestraat 49, Leuven, B-3000, Belgium; 3 Research Centre for Pharmaceutical Care & Pharmaco-economics, Faculty of Pharmaceutical Sciences, KU Leuven, Herestraat 49, Leuven, B-3000, Belgium; 4 Department of Pharmacy, University Hospitals Leuven, Herestraat 49, Leuven, B-3000, Belgium; 5 Finance & Accounting Department of the Jessa Hospital Hasselt, Stadsomvaart 11, Hasselt, B-3500, Belgium; 6 Medical Intensive Care Unit, University Hospitals Leuven, Herestraat 49, Leuven, B-3000, Belgium; 7 Department of Anesthesia & Intensive Care of the Jessa Hospital Hasselt, Stadsomvaart 11, Hasselt, B-3500, Belgium

## Abstract

**Introduction:**

The EPaNIC randomized controlled multicentre trial showed that postponing initiation of parenteral nutrition (PN) in ICU-patients to beyond the first week (Late-PN) enhanced recovery, as compared with Early-PN. This was mediated by fewer infections, accelerated recovery from organ failure and reduced duration of hospitalization. Now, the trial's preplanned cost analysis (N = 4640) from the Belgian healthcare payers' perspective is reported.

**Methods:**

Cost data were retrieved from individual patient invoices. Undiscounted total healthcare costs were calculated for the index hospital stay. A cost tree based on acquisition of new infections and on prolonged length-of-stay was constructed. Contribution of 8 cost categories to total hospitalization costs was analyzed. The origin of drug costs was clarified in detail through the Anatomical Therapeutic Chemical (ATC) classification system. The potential impact of Early-PN on total hospitalization costs in other healthcare systems was explored in a sensitivity analysis.

**Results:**

ICU-patients developing new infection (24.4%) were responsible for 42.7% of total costs, while ICU-patients staying beyond one week (24.3%) accounted for 43.3% of total costs. Pharmacy-related costs represented 30% of total hospitalization costs and were increased by Early-PN (+608.00 EUR/patient, p = 0.01). Notably, costs for ATC-J (anti-infective agents) (+227.00 EUR/patient, p = 0.02) and ATC-B (comprising PN) (+220.00 EUR/patient, p = 0.006) drugs were increased by Early-PN. Sensitivity analysis revealed a mean total cost increase of 1,210.00 EUR/patient (p = 0.02) by Early-PN, when incorporating the full PN costs.

**Conclusions:**

The increased costs by Early-PN were mainly pharmacy-related and explained by higher expenditures for PN and anti-infective agents. The use of Early-PN in critically ill patients can thus not be recommended for both clinical (no benefit) and cost-related reasons.

**Trial registration:**

ClinicalTrials.gov NCT00512122.

## Introduction

Previous non-conclusive trials investigating clinical effects of parenteral nutrition (PN) supplementing enteral nutrition (EN) in critically ill patients were small [1], and studies analyzing the resource use or costs associated with such nutritional strategies are scarce [2]. The Belgian multicenter EPaNIC (Impact of Early Parenteral Nutrition completing enteral nutrition In Critical illness) randomized controlled trial (RCT) has shown that withholding PN during the first week of critical illness (Late PN) was clinically superior to early supplementation of insufficient EN with PN (Early PN) [3]. In this study, 4,640 patients from seven intensive care units (ICUs) in three departments from two hospitals were randomly assigned to Early or Late PN. This trial had adequate statistical power to detect a difference in length of stay in the ICU and 90-day mortality, which are, respectively, the primary efficacy and safety endpoints [4-6]. Patients randomly assigned to the Early-PN group (n = 2,312) received intravenous glucose 20% on days 1 and 2, followed by PN, which was targeted to ± 100% of the caloric goal via combined EN and PN. When clinicians predicted that the patient would tolerate sufficient EN or oral feeds the following day, PN was not initiated. Patients randomly assigned to the Late-PN group (n = 2,328) received no PN during the first week in the ICU. In both groups, management of EN was identical and patients received parenteral trace elements, minerals, and vitamins early. Late PN proved to be safe as ICU, hospital, and 90-day mortality and feeding-related complications in the two groups were comparable. Moreover, Late PN reduced the incidence of new infections in the ICU, shortened the duration of organ support, and reduced ICU stay. Late PN also shortened hospital stay without affecting functionality at hospital discharge. A formal cost-effectiveness analysis was a preplanned substudy of the EPaNIC clinical trial [6]. As the EPaNIC study did not show a benefit for the more expensive nutritional strategy of Early PN, this preplanned analysis was futile. Hence, we present a cost analysis from the health-care payers' perspective, exploring the magnitude and the drivers of the cost difference that resulted from the Early PN nutritional strategy [6].

## Materials and methods

### Preliminary comments

Within the context of the change in the plan, the following analyses were performed: First, we analyzed the total health-care costs, dichotomized by the major clinical outcomes of the study, in order to generate insight into the relative contribution of patient outcomes to the cost differences between both groups. These two major clinical outcome variables were the acquisition of a new infection and a prolonged ICU stay. Second, eight cost categories, representing the different reimbursed services and products during ICU and hospital stays, were studied. The importance of each category to total hospitalization costs and the influence of Early versus Late PN were compared [7]. Third, the nature of the drugs responsible for differences in pharmacy costs was investigated by using the Anatomical Therapeutic Chemical (ATC) classification, the World Health Organization (WHO) tool for drug utilization research. Finally, a model was constructed to estimate the cost difference between Early and Late PN if all PN purchase costs would have been chargeable. Also, the total costs in the ICU for vitamins and insulin - as crucial components of the nutritional strategy - were described. The Institutional Review Board of the University Hospitals of the KU Leuven and of the Jessa Hospital approved the study protocol, including the health economy analysis, and all informed consent forms (ML4190).

### Data collection

The cost study design took into account previously published methodological guidelines [8-10]. For the construction of the cost tree and for the examination of cost categories, all direct health-care costs were calculated by using a bottom-up approach and were reported in euros, rounded at zero decimals. The cost data were not inflation-adjusted. During the trial, financial and drug utilization data were collected and verified automatically by the billing and warehousing collaborators according to their standard operating procedures. These collaborators were unaware of the ongoing trial. Upon completion of the EPaNIC study, the data warehousing collaborators were given only patient numbers, without the randomization group. All costs were retrieved from the individual patient invoices simultaneously for the entire study population by using the data warehousing system in the participating hospitals. These cost data were stored and processed in FileMaker Pro 8.0 (FileMaker Inc., Santa Clara, CA, USA). After data checks, only the clinical investigators (SV, MPC, and DM) were unblinded to treatment allocation.

No distinction was made between costs borne by the health insurance and the individual patient co-payments. Only direct health-care expenses, reflected by the official, nationally fixed, tariffs were included. The perspective of the health-care payer is recommended in the Belgian pharmaco-economic guidelines [11] and allows a precise cost data analysis based on patient invoices. Because the health-care payers' perspective was taken, the cost comparison of both arms of the study is inevitably influenced by the particularities of the reimbursement system in Belgian hospitals.

The relevant particularities for the study are the following:

• Medical services for which no fee can be charged (either to the patient or the insurance company) are not included in the invoice. Consequently, the costs for these services were not accounted for in this cost analysis.

• Some medical services (for example, clinical biology), independently of whether they are provided to the patient, are reimbursed largely through lump-sum payments. These medical services are billed on the invoice through per admission and per diem (PD) fees.

• A number of pharmaceutical products, including PN, are reimbursed in a 'mixed system': 25% of the list price is reimbursed in addition to a lump-sum reimbursement per hospital admission. Hence, only 25% of the PN cost is captured through the patient invoice.

• To cover the costs associated with the hospital stay (nursing, support services, and care products other than pharmaceutical products), Belgian hospitals receive an annual budget. 'Transfer' of this budget to the hospital is organized mainly (80%) through monthly cash advances and, to a small extent (20%), through a per admission and a PD fee. The levels of these fees are identical for all patients in a specific hospital. Hence, the 'per diem hospitalization costs' component of the patient invoice only partially reflects the true resource use by ICU patients.

Because patients were recruited between August 2007 and November 2010, invoices are based on price rates for four different years. During these four fiscal years, the number of patients randomly assigned to one of the two treatment groups did not differ. Since price fluctuations of measured cost elements are relatively small, no adjustment was made for the price variations during the course of the clinical study.

The in-depth cost analysis involved all patients from the original EPaNIC study, except 22 patients for whom the sum of the invoice details was different from the total amount invoiced. In order to avoid bias, these patients were excluded from the analysis before unblinding. As the study intervention took place only during ICU stay but costs may be affected after ICU discharge, the costs were examined in two periods. Period 1 extended from the day of ICU admission to the day of discharge from the ICU. Period 2 covered costs from after ICU discharge until the day of discharge from the hospital (not shown in Tables 1 and 2). The time horizon of this health economic evaluation was the entire index stay (that is, the hospital where the patient was initially included in the trial). Therefore, opportunity costs and costs generated in hospitals or rehabilitation centers, to which the patient was referred from the index hospital, were not taken into account. Since the time horizon was less than one year, neither costs nor clinical effects were discounted. As the EPaNIC trial was *a priori *powered only for clinical outcome variables and the number of patients available for the cost analysis was determined by the primary RCT, the statistical power of the trial to detect the observed difference in total costs was calculated *a posteriori *[12].

**Table 1:** Healthcare costs split by major cost categories

Category	Early PN, euros		Late PN, euros		Mean difference, euros	*P *value (*t *test)
			
	Mean	p25-p75	Mean	p25-p75		
Total hospital stay						
Honoraria	7,058.00	4,762.00-7,126.00	6,812.00	4,732.00-7,037.00	246.00	0.16
Pharmacy	5,478.00	1,871.00-5,835.00	4,870.00	1,875.00-5,548.00	608.00	0.01
Hospitalization costs (PD)	2,221.00	654.00-1,950.00	2,126.00	653.00-1,849.00	95.00	0.63
Blood products	1,345.00	204.00-1,517.00	1,290.00	204.00-1,523.00	56.00	0.43
Clinical chemistry	963.00	253.00-1,141.00	889.00	255.00-1,035.00	75.00	0.05
Radiology	550.00	167.00-665.00	519.00	1,688.00-625.00	30.00	0.11
Miscellaneous	184.00	0.00-105.00	178.00	0.00-124.00	7.00	0.60
Graft products	165.00	0.00-0.00	167.00	0.00-0.00	−1.00	0.96
Total	17,965.00	8,746.00-18,661.00	16,851.00	8,788.00-17,749.00	1,114.00	0.04
Period 1 (ICU)						
Honoraria	5,969.00	4,198.00-6,132.00	5,783.00	4,113.00-6,140.00	186.00	0.19
Pharmacy	4,679.00	1,754.00-5,210.00	4,231.00	1,737.00-5,175.00	448.00	0.04
Hospitalization costs (PD)	1,002.00	173.00-923.00	960.00	177.00-854.00	42.00	0.68
Blood products	1,216.00	167.00-1,351.00	1,125.00	166.00-1,383.00	91.00	0.17
Clinical chemistry	730.00	194.00-816.00	664.00	201.00-728.00	66.00	0.03
Radiology	332.00	61.00-400.00	310.00	64.00-338.00	23.00	0.12
Miscellaneous	52.00	0.00-0.00	51.00	0.00-0.00	1.00	0.86
Graft products	145.00	0.00-0.00	156.00	0.00-0.00	−11.00	0.73
Total	14,124.00	7,240.00-14,821.00	13,280.00	7,173.00-14,210.00	844.00	0.05

Early PN, parenteral nutrition administered during the first week of critical illness when enteral nutrition is insufficient; ICU, intensive care unit; Late PN, no parenteral nutrition administered before day 8 of critical illness; p25-p75, interquartile range (25th and 75th percentiles); PD, per diem.

**Table 2:** Pharmacie costs split by Anatomical Therapeutic Chemical classification system classes

Category	Early PN, euros		Late PN, euros		Mean difference, euros	*P *value (*t *test)
			
	Mean	p25-p75	Mean	p25-p75		
Total hospital stay						
0	618.00	1.00-239.00	585.00	1.00-207.00	34.00	0.71
A	134.00	25.00-146.00	124.00	24.00-126.00	9.00	0.14
B	1,162.00	202.00-1,102.00	942.00	176.00-830.00	220.00	0.006
C	137.00	16.00-119.00	133.00	16.00-109.00	5.00	0.61
D	23.00	0.00-13.00	17.00	0.00-12.00	6.00	0.12
G	3.00	0.00-0.00	7.00	0.00-0.00	−4.00	0.08
H	94.00	0.00-15.00	80.00	0.00-11.00	14.00	0.50
J	1,117.00	17.00-739.00	889.00	17.00-649.00	227.00	0.02
L	187.00	0.00-0.00	161.00	0.00-0.00	26.00	0.23
M	27.00	0.00-16.00	20.00	0.00-15.00	7.00	0.05
N	260.00	56.00-215.00	240.00	54.00-202.00	20.00	0.34
P	0.00	0.00-0.00	0.00	0.00-0.00	0.00	0.17
R	17.00	0.00-16.00	16.00	0.00-16.00	1.00	0.42
S	2.00	0.00-0.00	1.00	0.00-0.00	0.00	0.14
V	59.00	2.00-40.00	51.00	3.00-32.00	8.00	0.13
Total	3,843.00	414.00-3,866.00	3,271.00	397.00-3,314.00	572.00	0.01
Period 1 (in ICU)						
0	330.00	0.00-91.00	370.00	0.00-69.00	−40.00	0.60
A	90.00	16.00-92.00	82.00	14.00-74.00	8.00	0.11
B	797.00	133.00-718.00	612.00	101.00-467.00	186.00	0.0001
C	110.00	5.00-92.00	102.00	5.00-77.00	8.00	0.34
D	13.00	0.00-6.00	8.00	0.00-4.00	5.00	0.13
G	2.00	0.00-0.00	2.00	0.00-0.00	0.00	0.56
H	45.00	0.00-7.00	35.00	0.00-4.00	10.00	0.17
J	665.00	9.00-317.00	520.00	9.00-221.00	145.00	0.04
L	115.00	0.00-0.00	99.00	0.00-0.00	16.00	0.29
M	18.00	0.00-9.00	15.00	0.00-8.00	4.00	0.08
N	200.00	35.00-154.00	184.00	33.00-138.00	16.00	0.36
P	0.00	0.00-0.00	0.00	0.00-0.00	0.00	0.71
R	7.00	0.00-4.00	6.00	0.00-3.00	1.00	0.48
S	1.00	0.00-0.00	1.00	0.00-0.00	0.00	0.46
V	38.00	2.00-15.00	31.00	2.00-12.00	6.00	0.15
Total	2,435.00	252.00-2,005.00	2,071.00	235.00-1,458.00	364.00	0.03

Early PN, parenteral nutrition administered during the first week of critical illness when enteral nutrition is insufficient; ICU, intensive care unit; Late PN, no parenteral nutrition administered before day 8 of critical illness; p25-p75, interquartile range (25th and 75th percentiles).

### Cost tree

For further interpretation of the clinical effects in relation to costs, a cost tree was built [13,14]. As it was unaffected by treatment allocation, hospital mortality was not included in the cost tree. Therefore, the cost tree was constructed on the treatment allocation (first branch) and established excess cost-generating events that were affected by the treatment allocation: new infection [15,16] (second branch) and prolonged critical illness as defined by an ICU stay beyond 8 days [17] (third branch). The acquisition of a new infection and ICU stay beyond 8 days were thus chosen as branches because they were the principal acute clinical findings of the EPaNIC trial [1]. For each branch, the proportion of the total patient population meeting the branches' clinical criteria, the total health-care costs, and the mean total health-care costs per patient were reported.

### Examination of cost categories

So that the origin of the total cost difference between Early and Late PN could be clarified comprehensively, a second export for the detailed costs of all 4,640 patients was done. These costs were allocated to one of eight categories, a common classification used for hospital invoices in Belgium: PD hospitalization costs, honoraria for medical and allied health-care services, pharmacy costs, blood products, clinical chemistry, radiology, graft products, and a miscellaneous category [7]. However, during a check of the blinded exports for total hospitalization costs and the detailed export of costs per category, inconsistency was detected in 22 patients. So that bias could be avoided, this 0.47% of patients was deleted from the detailed analyses and so data retrieval did not need to be requested at the single patient level. The different cost categories were reported for periods 1 and 2 (not shown in Tables 1 and 2) separately and were aggregated. A Pareto chart visualized the contribution of each category to total health-care costs [18]. Cost drivers were defined as the combination of the largest cost categories that explained at least 80% of the total health-care costs in the Early-PN group. For these eight categories, cost differences between both study arms during both periods were assessed.

### Drug costs

So that the nature of the drugs responsible for cost differences between Early and Late PN could be identified, drug costs were analyzed by using the ATC classification system, the tool for presenting drug utilization statistics from the WHO [19] (Table 3). Drugs are classified in groups at five different levels. The drugs are divided into 14 main groups (first, or 'anatomical', level) and into pharmacological/therapeutic subgroups (second level). The third and fourth levels are chemical/pharmacological/therapeutic subgroups, and the fifth level is the chemical substance. The billed units for each drug (level 5 of the ATC classification) were retrieved from the data warehousing system from the pharmacies of the participating hospitals. The model, estimating the drug costs for every ATC class, used the official list prices of 2011. The total pharmacy costs mentioned previously were the sum of drug costs billed in their respective fiscal year. Devices or appliances accounted for in the data warehousing system, but not ATC-classified (for example, disposables, drains, and wound dressings), were grouped under the ATC-0 code. So that the highest accuracy could be provided, insulin costs were based on units administered per patient during ICU stay as recorded in the clinical research file.

**Table 3:** Anatomical Therapeutic Chemical classification system: level 1

Code	Contents
A	Alimentary tract and metabolism

B	Blood and blood-forming organs

C	Cardiovascular system

D	Dermatologicals

G	Genito-urinary system and sex hormones

H	Systemic hormonal preparations, excluding sex hormones and insulins

J	Anti-infectives for systemic use

L	Antineoplastic and immunomodulating agents

M	Musculo-skeletal system

N	Nervous system

P	Antiparasitic products, insecticides, and repellents

R	Respiratory system

S	Sensory organs

V	Various

Assessed on [19].

### Cost evaluation and sensitivity analysis

In the Belgian health-care system, the patients' invoices charge only 25% of PN purchase costs and thus unavoidably underestimate the cost of Early PN in the ICU. Therefore, in the sensitivity analysis, the impact of PN on the total incremental health care costs was calculated as if all PN purchase costs were chargeable to the health-care payer. We therefore added 75% of the mean costs during ICU stay in ATC subcategory level 4 B05BA (PN) to the total mean incremental health-care costs obtained from the patient invoices.

### Statistical analysis

All costs are presented as the mean and the interquartile range (25th and 75th percentiles). The costs in the two treatment groups were analyzed with the Student *t *test, and the costs between periods 1 and 2 were analyzed with the paired *t *test [12,20]. *P *values of 0.05 or lower were deemed significant. Analyses were performed with JMP (version 8.0.1) (SAS Institute Inc., Cary, NC, USA). All analyses were performed on an intention-to-treat basis.

## Results

### Summary of clinical results and total health-care costs

As previously reported, Late PN prevented new infections in the ICU and enhanced organ function recovery. Moreover, Late PN, in comparison with Early PN, shortened ICU and hospital stays and reduced mean total incremental health-care costs by €1,110.00 [3]. Calculation (with a one-tail test using an alpha level of 5%) of the statistical power of the observed overall difference in total incremental costs - Early PN (n = 2,312, mean cost of €17,973.00, standard deviation (SD) of €18,965.00) and Late PN (n = 2,328, mean cost of €16,863.00, SD of €18,190.00) - yielded a value of 65.2% [21].

### Cost tree

**Figure 1:**
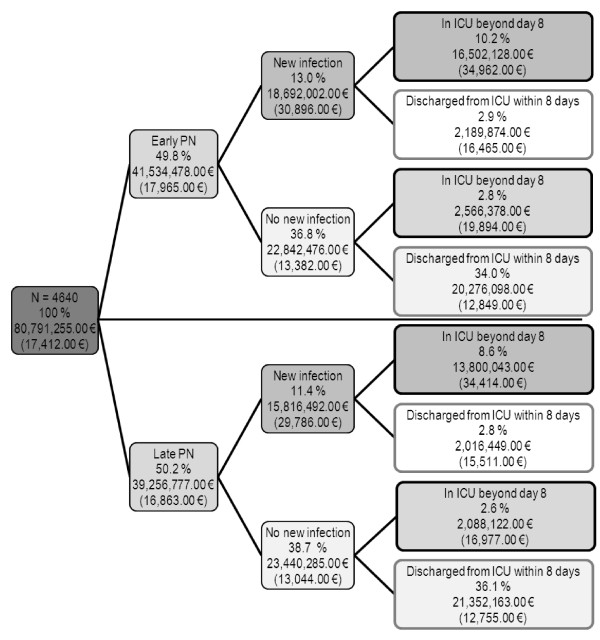
**Cost tree (cost allocation in the form of a decision tree)**. Costs are allocated by studied randomly assigned treatment (first branch), by the acquisition of a new infection (second branch), and by prolonged intensive care unit (ICU) stay, defined as an ICU stay beyond 8 days (third branch). The percentages represent the proportion of patients for the entire study population in each branch. The costs in euros (EUR) represent the total costs for all patients in each branch and - between brackets - mean cost per patient. Costs reported in euros are rounded at zero decimals. Early PN, parenteral nutrition administered during the first week of critical illness when enteral nutrition is insufficient; Late PN, no parenteral nutrition administered before day 8 of critical illness.

Only 24.5% of the patients acquired a new infection in the ICU but accounted for 42.7% of the total costs during the EPaNIC study (Figure 1). Likewise, patients with a prolonged ICU stay accounted for 43.3% of the total costs but represented only 24.2% of the entire population. Late PN reduced mean total costs per patient in all branches of the tree. In the clinically strived for patient population (70.1% of the total population), namely patients who do not develop a new infection and who do not require prolonged ICU support, the cost increase by Early PN was only €94.00 per patient. In contrast, in patients who had a prolonged ICU stay without a new infection, the mean cost increase by Early PN was €2,917.00.

### Examination of cost categories

**Figure 2:**
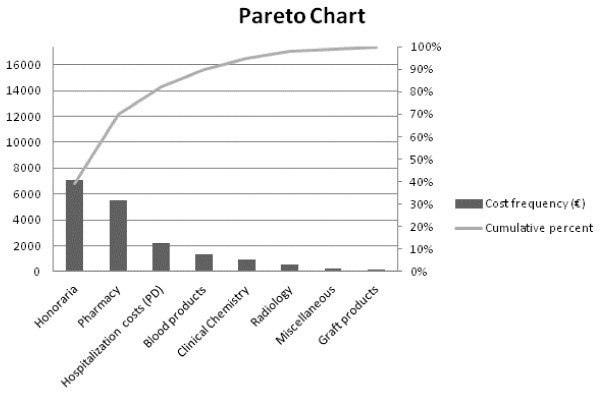
**Pareto chart of the costs for the total EPaNIC trial population**. The histogram depicts the relative size of each cost category in comparison with the total cost. The line graph shows their cumulative contribution. Important cost drivers for total hospital stay in the EPaNIC trial population are highlighted. EPaNIC, Impact of Early Parenteral Nutrition completing enteral nutrition In Critical illness; PD, per diem.

For the total hospital stay, honoraria, pharmacy, and PD hospitalization costs represented 82.1% of the total costs (Figure 2 and Table 1). For period 1 (ICU), honoraria, pharmacy, and blood products represented 84.0% of the total costs; 80.9% of the total costs in period 2 (after ICU) were driven by PD hospitalization costs, honoraria, and pharmacy costs (data not shown).

The overall increased cost for the Early-PN group was brought about by higher costs for pharmacy and for clinical chemistry. Those cost differences were significantly present only in period 1, which was responsible for 79% of the total hospital stay costs. The increased pharmacy costs with Early PN (mean difference + €608.00 per patient, *P *= 0.01) explained 54.6% of the mean total difference in costs between the two randomization groups (+ €1,114.00 per patient).

### Drug costs

The difference in pharmacy costs was explained predominantly by the fact that Early PN increased expenses in the ATC level 1 categories B (blood products and perfusion fluids) and J (anti-infective agents for systemic use) and by a smaller difference in category M (musculo-skeletal system) (Table 2). Costs for drug utilization in the ICU represented 63.6% of the total drug costs. Total costs in ATC subcategory B05BA 'Parenteral nutrition' (level 4) during ICU stay were €610,584.00 in the Early-PN group (mean of €266.00 per patient) and €317,275.00 in the Late-PN group (mean of €137.00 per patient); the mean difference was €129.00 per patient (*P *<0.0001). The largest proportions of these PN costs were covered by combined PN bags (B05BA10: 88.7% in the Early-PN group and 80.4% in the Late-PN group) followed by carbohydrate solutions (B05BA03: 8.9% in the Early-PN group and 16.1% in the Late-PN group). The costs for separate protein (B05BA01) or lipid solutions (B05BA02) were negligible in both groups.

Total costs for vitamins were €65,162.00 (mean of €28.00 per patient) and €49,981.00 (€21.00 per patient) in the Early- and the Late-PN group, respectively. Total costs for insulin, based on units administered per patient, were €32,230.00 (mean of €14.00 per patient) in the Early-PN group and €20,392.00 (mean of €9.00 per patient) in the Late-PN group.

Aggregated costs of ATC level 2 subcategories J01 'Antibacterials for systemic use' and J02 'Antimycotics for systemic use' during ICU stay were €1,363,396.00 in the Early-PN group (mean of €593.00 per patient) and €1,026,189.00 in the Late-PN group (mean of €443.00 per patient); the mean difference was €150.00 per patient (*P *= 0.26). Class B and J drugs together represented 59.3% of the total pharmacy costs in the Early-PN group and 56.0% in the Late-PN group.

### Cost evaluation and sensitivity analysis

Adding 75% (not billable fraction) of the PN purchase costs obtained from the pharmacy warehouse (ATC B05BA) for period 1 to the total costs in both groups resulted in mean total costs of €18,173.00 [(€266.00 × 0.75) + €17,973.00] in the Early-PN group and €16,963.00 [(€137.00 × 0.75) + €16,863.00] in the Late-PN group, increasing the mean difference to €1,210.00 per patient (*P *= 0.02).

## Discussion

A large Belgian multicenter RCT showed that Late PN prevented infections and enhanced recovery in a mixed population of critically ill patients [3]. Moreover, Late PN resulted in lower total billed costs during hospital stay. As Early PN did not demonstrate a clinical benefit and is more expensive, a formal cost-effectiveness analysis was superfluous. Therefore, in this health economy analysis, we sought to further investigate this mean cost increase of €1,110.00 per patient by Early PN. The cost-tree analysis confirmed existing literature that the development of new infections and prolonged ICU stay have a major impact on acute health-care costs for critically ill patients [15,17]. Patients who contracted a new infection in the ICU or had to stay in the ICU beyond 8 days each represented a quarter of the ICU population but almost half of the costs.

Early PN consistently increased mean health-care costs per patient in all patient subpopulations. Honoraria, pharmacy costs, and PD hospitalization cost categories represented over 80% of the total hospital stay costs. During ICU stay, costs for blood products also contributed importantly to total costs. This is in line with a recent micro-costing study in which the requirement for expensive interventions, comprising blood products, antifungals, and hemofiltration, was the main driver for ICU costs [7]. In the ICU, Early PN increased costs for the large pharmacy cost category and for clinical chemistry. The lower proportion of costs and the considerable individual cost variation resulted in a blunted effect of Late PN after ICU discharge. The higher pharmacy costs in the Early-PN group accounted for over 50% of the cost increase. Not only the cost for ATC class B (drugs for blood and blood forming organs) -which includes PN- but also the costs for ATC class J medication (systemic antibacterial and antimycotic agents) were higher. The higher costs for antibiotics and antimyotics corroborate the finding from the clinical study that Early PN increased the incidence of new infections.

More prescription of combined PN bags was the single most important contributor to the costs for PN. The costs for vitamins and insulin, generally regarded as part of the nutritional strategy, were much lower. Pharmacy costs obtained from the accounting department were higher than the costs for drugs retrieved from the pharmacy data warehousing system. The costs for preparation of drugs and their delivery may have contributed to this difference. In addition, the costs for non-ATC-classified pharmacy products, such as disposables, were not entirely covered in the pharmacy data warehousing system. Moreover, the latter model used 2011 prices for all patients whereas the accounting department provided true costs per fiscal year. The sensitivity analysis making total incremental health-care costs comparable with other health-care systems that fully charge PN revealed a mean €1,210.00 cost difference per patient, benefiting Late PN.

From the EPaNIC RCT it can be concluded that the nutritional strategy of Early PN was inferior to Late PN in adult critically ill patients. Early PN was less effective (605 versus 531 patients with a new ICU infection and prolonged duration of organ replacing therapy and of ICU and hospital dependency) and more expensive (€610,584.00 versus €317,275.00) than Late PN.

In light of the lack of clearly demonstrated benefits of PN, clinicians considering its early administration should realize that, besides the purchase cost of PN itself, this strategy will, above all, generate important expenses mediated by clinical complications. The benefit of Late PN, from clinical and cost perspectives, places the practice of withholding PN until day 8 in the ICU in the southeast quadrant of the cost-effectiveness plane [14]. Rarely, intervention studies in hospitalized patients bring forward strategies that are less costly and at the same time more effective than standard practice. The strategy of blood glucose control with intensive insulin therapy, which reduces morbidity and mortality in surgical critically ill patients and has a low technology cost of €72.00 per patient, also resulted in a large cost saving of €2,638.00 per patient [22]. Other studies in a broad spectrum of critically ill patients confirmed these economic benefits [23,24]. In contrast, strategies depending on newly developed drugs are usually more costly. A notable example is the use of activated protein C (dotrecogin alpha), which appeared to be effective only in patients with very severe sepsis but which costs US $160,000.00 per life saved [25,26].

This health economic analysis of the EPaNIC trial has some shortcomings that should be highlighted [27]. As the economic evaluation is trial-based, the occurrence of a protocol-driven resource use cannot be excluded and this could reduce generalizability. The micro-costing approach may have further amplified this lack of generalizability but contributed to the validity of cost estimates. Also, although the EPaNIC trial was adequately powered for the clinical outcomes, its statistical power to detect differences in total costs was somewhat weaker because of the substantial variability in total costs per patient. Lastly, the economic evaluation was set in the Belgian health-care system. Hence, cost impact of Early PN cannot be generalized as such to other health-care systems. Although the actual cost savings will differ from hospital to hospital and from country to country, the beneficial cost effect of Late PN is likely to be confirmed as long as the clinical data are confirmed in other settings.

## Conclusions

This health economy analysis based on accounting department and pharmacy warehouse data provides external validation of the clinical results of the EPaNIC trial. It is clear that the practice of Early PN in critically ill patients cannot be recommended as it did not show benefit and it increased health-care costs.

## Key messages

• Early parenteral nutrition (PN) increased total health-care costs by increasing pharmacy-related costs, which represent 30% of the total costs from a health-care payer's perspective.

• Early PN increased pharmacy-related costs not only by PN purchase costs but also by high increases in the costs for anti-infective agents.

• This health economy analysis based on accounting department and pharmacy warehouse data provided external validation of the clinical results of the EPaNIC trial.

• For clinical and for health economic reasons, early PN in critically ill patients cannot be recommended.

## Abbreviations

ATC: Anatomical Therapeutic Chemical; Early PN: parenteral nutrition administered during the first week of critical illness when enteral nutrition is insufficient; EN: enteral nutrition; EPaNIC: Impact of Early Parenteral Nutrition completing enteral nutrition In Critical illness; ICU: intensive care unit; Late PN: no parenteral nutrition administered before day 8 of critical illness; PD: per diem; PN: parenteral nutrition; RCT: randomized controlled trial; SD: standard deviation; WHO: World Health Organization.

## Competing interests

The KU Leuven received an unconditional and non-restrictive research grant for the EPaNIC trial from Baxter Healthcare SAS(Maurepas, France).

## Authors' contributions

SV (joint first author) contributed to the design, acquisition, analysis, and interpretation of the data and the drafting of the manuscript. MPC (joint first author) and GVdB contributed to the conception, design, analysis, and interpretation of the data and the drafting of the manuscript. KK, SS, JC, AW, and JD contributed to the analysis and interpretation of the data and the critical revision of the manuscript for important critical content. TDR, GP, PJW, TG, KP, and AM contributed to the acquisition of the data and the critical revision of the manuscript for important critical content. DM contributed to the conception, design, acquisition, analysis, and interpretation of the data and the drafting of the manuscript. All authors read and approved the final manuscript.
